# Lactancia materna y alojamiento en el abordaje del síndrome de abstinencia neonatal. Revisión panorámica

**DOI:** 10.23938/ASSN.1048

**Published:** 2023-08-28

**Authors:** Paula Baeza-Gozalo, Sara Sola-Cía, Olga López-Dicastillo

**Affiliations:** 1 Universidad Pública de Navarra Universidad Pública de Navarra-UPNA Facultad de Ciencias de la Salud Departamento de Ciencias de la Salud Pamplona Spain; 2 Servicio Navarro de Salud-Osasunbidea Hospital Universitario de Navarra Servicio Navarro de Salud- Osasunbidea Pamplona Spain; 3 Research Group CreaP. Pamplona. España. Research Group CreaP Pamplona España; 4 Instituto de Investigación Sanitaria de Navarra (IdiSNA) Pamplona España

**Keywords:** Síndrome de abstinencia neonatal, Neonato, Lactancia materna, Alojamiento conjunto, Neonatal abstinence syndrome, Newborn, Breastfeeding, Rooming-in

## Abstract

En la actualidad, el aumento del consumo de drogas en gestantes está provocando un incremento del síndrome de abstinencia neonatal (SAN). El abordaje de dicho síndrome varía en la práctica clínica y, en algunos centros, se tiende a suspender tanto la lactancia materna como el alojamiento conjunto. El objetivo de este trabajo es analizar los efectos de la lactancia materna y el alojamiento conjunto en neonatos con SAN mediante la realización de una revisión panorámica de trabajos publicados en PubMed y CINAHL.

Según los once trabajos incluidos, tanto la lactancia materna como el alojamiento conjunto reducen la estancia hospitalaria, así como la necesidad y la duración del tratamiento farmacológico. Además, el alojamiento conjunto disminuye la probabilidad de admisión en Cuidados Intensivos Neonatales, aunque no mejoró la severidad de los signos del SAN. Los bebes alimentados con lactancia materna presentaron signos significativamente más leves de abstinencia y, una mayor probabilidad, aunque no significativa, de ser reingresados. No hay evidencia de que el alojamiento conjunto disminuya la readmisión hospitalaria tras el alta.

Los hallazgos justifican que tanto la cohabitación como la lactancia se deberían mantener siempre que sea posible en el abordaje de este síndrome para no empeorar las condiciones del neonato, tomando las medidas oportunas para garantizar la seguridad del niño y de la madre.

## Abstract (en)

At present, more babies are born with neonatal abstinence syndrome due to the increased use of certain substances by their mothers while pregnant. The therapeutic approaches for this syndrome vary in clinical practice, and in some centres, breastfeeding and rooming-in are interrupted. The aim of this work was to analyse the effects of breastfeeding and rooming-in in infants with neonatal abstinence syndrome by conducting a scoping review of the related literature in PubMed and CINAHL.

Eleven papers were included, which showed that breastfeeding and rooming-in reduced hospital stay and the need for and duration of pharmacological treatment. In addition, rooming-in decreased the likelihood of admission to the Neonatal Intensive Care Unit, although there was no improvement of the severity of neonatal abstinence syndrome signs. Breastfed infants had milder withdrawal signs and, although they were more likely to be readmitted than formula-fed infants were, there were no statistically significant differences. There was no evidence that rooming-in decreased hospital readmission after discharge.

Our findings support the maintenance of cohabitation and breastfeeding whenever possible in the management of this neonatal abstinence syndrome, so as not to worsen the conditions of the neonate, while introducing measures to ensure mother-child safety.

## INTRODUCCIÓN

El síndrome de abstinencia neonatal (SAN) se define como el conjunto de manifestaciones clínicas que expresa el recién nacido cuya madre es consumidora de sustancias psicotrópicas como la cocaína, la marihuana y los opioides^1^. Se produce fundamentalmente desde las primeras 24 horas del nacimiento hasta el tercer día de vida. Entre las manifestaciones más comunes destacan la irritabilidad, la hipertonía, el temblor, la sudoración, la hipertermia, los bostezos, la hiperfagia y los vómitos^1^^-^^4^. A todo ello se le añaden las consecuencias del consumo materno de drogas durante el embarazo, como la prematuridad y el bajo peso al nacer^1^. Además, los neonatos que presentan SAN tienen ingresos hospitalarios prolongados y más probabilidad de ingresar en la unidades de cuidados intensivos neonatales (UCIN) que los que no han estado expuestos a ningún tipo de droga durante el embarazo^5^.

El SAN se ha convertido en un gran problema en las últimas décadas, tanto a nivel nacional como mundial, ya que su tasa de diagnóstico se ha visto incrementada en un 300% desde 1999 hasta 2013^6^. Aunque hay pocos estudios que informen sobre su incidencia y prevalencia, en Estados Unidos la incidencia de SAN aumentó de 1,5 a 8 casos por cada 1.000 nacimientos entre 2004 y 2014^5^, y cifras más recientes la sitúan en torno al 6,7 por cada 1.000 nacimientos^7^.

En España, los datos indican que el 1,5% de las mujeres embarazadas consumen drogas ilegales, siendo la más frecuente el cannabis (0,3%)^8^. El 1,5% de los neonatos han podido estar expuestos a algún tipo de droga durante el periodo intrauterino y aproximadamente el 28% de ellos sufren SAN^8^^,^^9^. Cuando la adicción se identifica mediante la recogida de muestras biológicas en pares de mujeres y neonatos, el dato de consumo es más elevado que cuando es informado por las madres, encontrando consumos del 6,4% de cannabis, 8,3% de cocaína y 3,8% de heroína y metadona^10^, a pesar de haber identificado un descenso significativo del consumo a lo largo del embarazo^10^.

La prevención del SAN pasaría por evitar el consumo de drogas durante el embarazo. Sin embargo, cuando una gestante consume y el recién nacido presenta SAN, la literatura muestra una gran variabilidad en las prácticas asistenciales para su cuidado. Algunos centros y profesionales siguen desaconsejando la lactancia y separan inmediatamente a la diada madre-hijo por la incapacidad de las madres consumidoras de drogas para cuidar a su hijo, así como por la necesidad de monitorizar al neonato (tensión arterial, frecuencia cardiaca, frecuencia respiratoria y saturación de oxígeno, principalmente), evaluarlo con frecuencia y mantenerlo en observación al ser tratado con algún fármaco^11^^-^^15^. Sin embargo, estudios recientes muestran que tanto la lactancia como el alojamiento conjunto del niño con la madre (*rooming o rooming-in*) son medidas no farmacológicas que tienen un efecto positivo en el recién nacido con SAN^13^^,^^16^^,^^17^, fomentando la presencia y el compromiso materno con el cuidado y la atención del neonato^17^^-^^19^. La separación de la diada y la suspensión de la lactancia materna pueden tener consecuencias contrarias a las deseadas al empeorar el estado del neonato y su nivel de estrés, lo que aumenta la necesidad de medicación para el control del SAN^13^^,^^16^^,^^17^, además, de poner en riesgo el establecimiento del vínculo materno-filial^17^^-^^19^.

Por estos motivos, algunos hospitales y centros sanitarios han cambiado sus prácticas y apuestan por promocionar la lactancia materna y el alojamiento conjunto, permitiendo que las madres consumidoras y/o ex-consumidoras de sustancias ilícitas permanezcan continuamente con sus bebés durante toda la estancia^13^^,^^15^^,^^17^.

Ante los riesgos que la adopción de unas u otras medidas puede suponer para el neonato, el objetivo de esta revisión fue explorar los efectos que tienen la lactancia materna y el alojamiento conjunto en el recién nacido con SAN en seis variables: la duración de la estancia hospitalaria, la necesidad de tratamiento farmacológico, la duración del tratamiento farmacológico, la severidad de los signos del SAN, la admisión en las UCIN y la necesidad de reingreso hospitalario tras el alta. Esto puede ayudar a identificar prácticas asistenciales seguras en el cuidado del neonato con SAN y evitar aquellas que ponen en riesgo su salud.

## MATERIAL Y MÉTODOS

Se realizó una revisión panorámica de la literatura, que mapea la información existente acerca de un tema e identifica y explica diversos conceptos generales que lo sustentan^20^, en las bases de datos PubMed y CINAHL.

A fin de obtener información concreta y específica para responder al objetivo se realizó una búsqueda de información a partir de la estructura PICO (pacientes, intervención, comparación, resultado), cuyos términos de búsqueda se muestran en la Tabla 1:

- P (*patient*): neonatos con SAN/expuesto a drogas
- I (*intervention*): lactancia materna, alojamiento conjunto
- C (*comparison*): lactancia artificial, admisión en UCIN
- O (*outcome*): desarrollo, bienestar, relación, recuperación.

**Tabla 1:** Desarrollo de la pregunta PICO para la búsqueda en bases de datos

Población		Intervención		Comparación		Resultado
newborn with NAS	*AND*	breastfeeding	*AND*	neonatal intensive care unit	*AND*	attachment
*OR*		*OR*		*OR*		*OR*
infant with NAS		breast-feeding		NICU		bonding
*OR*		*OR*		*OR*		*OR*
neonatal abstinence syndrome		breast milk		infant formula		development
*OR*		*OR*		*OR*		*OR*
neonatal withdrawal syndrome		lactation		artificial lactation		wellbeing
*OR*		*OR*				*OR*
NAS		rooming-in				progress
*OR*						*OR*
substance expos*						length of stay
*OR*						*OR*
SEN						LOS
*OR*						*OR*
drug expos*						NAS severity
						*OR*
						recovery
						*OR*
						improvement

NAS: *neonatal abstinence syndrome*; SEN: *substance exposed newborn*; NICU: *neonatal intensive care unit*; LOS: *length of stay*. Se empleó el truncamiento de palabras para incluir posibles variaciones empleadas.

Se incluyeron artículos en español e inglés 1) sobre neonatos con SAN cuyas madres fuesen consumidoras de drogas ilegales y/o fármacos durante el embarazo, 2) que utilizaran tanto tratamientos farmacológicos como no farmacológicos en la atención del recién nacido con SAN, 3) que comparasen la lactancia materna con la de fórmula en el recién nacido de madres consumidoras de drogas, y 4) que comparasen el alojamiento conjunto con otras formas de alojamiento en el recién nacido de madres consumidoras de drogas. Se excluyeron las opiniones respecto al SAN y su abordaje, y los estudios que incluyesen únicamente lactancia artificial como modo de alimentación del recién nacido con SAN.

## RESULTADOS

**Figura 1:**
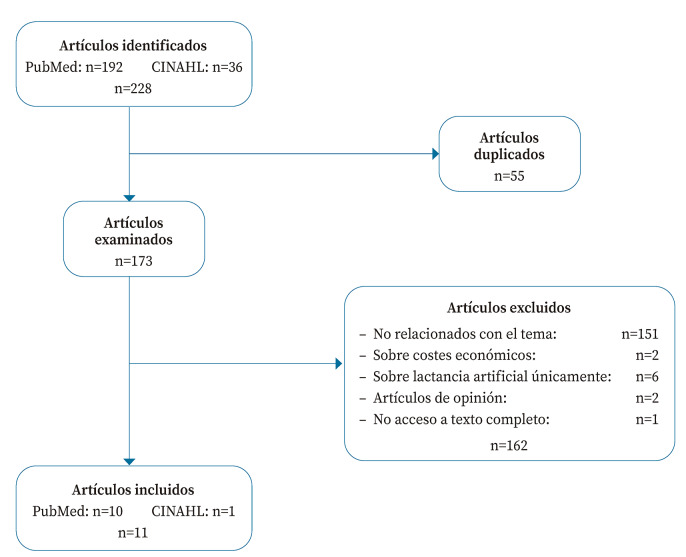
Diagrama resumen del proceso de selección de artículos.

La búsqueda en PubMed y CINAHL produjo 228 artículos, que se redujeron a 173 tras eliminar los duplicados. La aplicación de los criterios de selección permitió incluir once artículos^11^^,^^14^^-^^17^^,^^21^^-^^26^ en esta revisión (Fig. 1).

Las características de los once estudios se resumen en la Tabla 2. Todos ellos se realizaron en América del Norte: cinco en Canadá^11^^,^^15^^,^^22^^,^^23^^,^^25^ y seis en Estados Unidos^14^^,^^16^^,^^17^^,^^21^^,^^24^^,^^26^. Respecto al diseño, los estudios de cohortes fueron los más habituales^11^^,^^15^^,^^16^^,^^21^^,^^23^^,^^24^^,^^26^ (n=7; 63,6%) ; dos fueron estudios de intervención^14^^,^^25^ y los otros dos revisiones bibliográficas^17^^,^^22^.

Los estudios incluyeron 3.405 neonatos, con edad gestacional de 35 semanas o más; las dos revisiones no indicaron el tamaño muestral y un estudio no indicó la edad^23^. Los opiodes fueron el tipo de droga más consumida, especialmente la metadona (Tabla 2); dos estudios no especificaron las sustancias^11^^,^^16^. La severidad del SAN se evaluó mediante la escala de Finnegan^21^^,^^24^ o de Finnegan modificada^15^^,^^23^^,^^25^^,^^26^; tres artículos no especificaron la herramienta de medición^11^^,^^14^^,^^16^.

**Tabla 2:** Resumen de los artículos incluidos en la revisión panorámica

Autoría	Objetivo	Diseño	Resultados
Año		Fuente de datos	
País		Tamaño muestral	
		Tipo de droga	
		Medición del SAN	
Abrahams	Impacto del AC (respecto del CE) y de la educación prenatal en el bienestar y cuidado del neonato con SAN, y asociación con ingreso en UCIN y duración de la estancia.	- Cohortes retrospectivo	AC respecto de CE:
y col ^11^		- Madres consumidoras de sustancias de dos fuentes de datos:	- < ingreso en UCIN de bebés a término (23,5 vs 38,5, p=0,01)
2010		1) Programa de Salud Perinatal de la Columbia Británica. Indicador: *consumo de sustancias*	- < tiempo de estancia en UCIN (1,1 vs 3,1 días, p<0,001)
Canadá		2) ICD 10CA del Resumen de Altas del Instituto Canadiense de Información Sanitaria. Indicador: *consumo materno de drogas que afectan al embarazo* e *identificación de droga específica*.	- > tiempo de estancia (17,6 vs 8,1 días, p<0,001).
		- n=952 RN con SAN	
		355 AC (71,8% a término)	
		597 CE (73% a término)	
		- Sustancias ilícitas (n/e)	
		- n/e	
Cree y col^14^	Asociación del AC (respecto de UCIN) con la duración de la estancia (hospital aria, UCIN), la necesidad de iniciar tratamiento farmacológico para mejorar la abstinencia y su duración.	- Intervención, proyecto de mejora	AC respecto de UCIN:
2019		- Historia clínica	- < duración del ingreso (10,1 vs 14 días, p=0,014)
EEUU		- n=88 RN:	- < necesidad de tratamiento farmacológico (42,5 vs 52,1%, p=0,339)
		40 AC + cribado SAN	- < duración del tratamiento (9,71 vs 15,68 días, p=0, 023).
		48 UCIN	- < duración de estancia en UCIN (0,2 vs 8,2 días, p<0,001).
		- Metadona y buprenorfina	
		- n/e	
McKnight	Asociación del AC (respecto de UCIN) con necesidad de farmacoterapia y duración del ingreso y del tratamiento.	- Cohortes retrospectivo	AC respecto de UCIN:
y col^15^		- Historia clínica	- -< duración del ingreso (5 vs 24 días, p<0,001)
2016		- n=44 RN con SAN	- < necesidad de tratamiento farmacológico (15 vs 83,3%, p<0,001)
Canadá		20 AC	- < duración del tratamiento (24 vs 29 días, p=0,83).
		24 UCIN	
		- Metadona y otros opioides (n/e)	
		- Escala modificada de Finnegan.	
Favara y col^16^	Asociación del tipo de lactancia (cualquier tipo de LM y LA) con la duración del ingreso y del tratamiento farmacológico, y la tasa de readmisión.	- Cohortes retrospectivo	Cualquier tipo de LM (LME + LMx):
2019		- Optum Neonatal Database	- < duración del ingreso (19 vs 20 días, p=0,01
EEUU		- n=1.738 RN	- < duración del tratamiento (14 vs 17, p=0,04)
		430 LM (70 LM exclusiva, 360 LMx)	- > tasa de readmisión (1,6 vs 1,3%, p=0,3).
		1.308 LA	
		- Droga n/e	
		- n/e	
MacMillan	Comparar diferentes modelos de cuidados y técnicas y su asociación con sus resultados en el RN.	- Revisión	La LM, el AC y otras técnicas no farmacológicas deben ser la primera opción en el tratamiento de RN con síntomas de SAN, porque mejoran su bienestar, entre otros aspectos.
y col^17^		- n/e	
2019		- n/e	
EEUU		- Opioides	
		- -n/e	
Lembeck y col^21^	Asociación del tipo de lactancia (LM, LA estándar y LA baja en lactosa) con la duración de la estancia hospitalaria y del tratamiento farmacológico.	- Cohortes retrospectivo	La LM, en comparación con la LAe y LAbl (p<0,01):
2020		- Base de datos hospitalaria	- < estancia hospitalaria (7,4 y 10,3 días menos)
EEUU		- n=249 RN con SAN	- < duración tratamiento farmacéutico (6,9 y 10,8 días menos).
		65 LM	
		147 LAe	
		37 LAbl	
		- Metadona, buprenorfina, cocaína, heroína, benzodiacepinas, marihuana, anfetaminas	
		- Escala de Finnegan.	
McQueen	Asociación del tipo de lactancia (LM y LA) con la duración de la estancia hospitalaria, la necesidad de medicación, severidad de los síntomas de SAN y tiempo hasta su aparición.	- Revisión sistemática	LM respecto a LA:
y col^22^		- PubMed, CINAHL, Nursing and Allied Health, PyschINFO, Evidence Based Medicine, Web of Science, MEDLINE.	- < necesidad de medicación
2019		- n/e	- < duración de ingreso
Canadá		- Opioides (metadona o buprenorfina)	- <severidad del SAN
		- n/e	- > tiempo hasta aparición de los síntomas.
McQueen	Asociación del tipo de lactancia (LM, LMx, LA) con la severidad del SAN.	- Cohortes	LM respecto a LMx o LA:
y col^23^		- Historia clínica	- <severidad del SAN con LM (4,9) que con LMx o LA (6,5 y 6,9 puntos, p<0,001).
2011		- n=25 RN	
Canadá		8 LM	
		11 LMx	
		9 LA	
		- Metadona	
		- Escala modificada de Finnegan.	
Grossman	Asociación del tipo de ingreso (planta de hospitalización, UCIN o ambas unidades) con la duración de la estancia hospitalaria, la necesidad de farmacoterapia y las readmisiones hospitalarias.	- Cohortes	Planta frente a UCIN o ambas:
y col^24^		- Historia clínica	- <duración de la estancia (8,5 vs 23 o 18 días, p<0,001)
2020		- n=159 RN	- < necesidad de tratamiento (58 vs 84 o 94%, p<0,001)
EEUU		50 UCIN	- <tasa de readmisión (0 vs 3 o 0%, p=0,332).
		60 planta pediátrica	
		49 UCIN+planta	
		- Metadona	
		- Escala de Finnegan.	
Newman y col^25^	Asociación del AC (respecto de UCIN) con la duración del ingreso hospitalario, la necesidad de tratamiento farmacológico y la satisfacción de las madres.	- Estudio de intervención.	AC respecto de UCIN (p<0,001):
2015		- Historia clínica y encuestas de satisfacción a las madres.	- <duración del ingreso (7,9 vs 24,8 días)
Canadá		- n=45 RN	- <necesidad de tratamiento farmacológico (14,3 vs 83,3%)
		24 UCIN	- 100% de las madres valoró el programa de AC con una satisfacción de 4 sobre 5.
		21 AC	
		- Opioides	
		- Escala modificada de Finnegan.	
Singh y col^26^	Asociación del AC (presencia de los padres respecto de UCIN) con la necesidad de ingreso en UCIN, duración del ingreso hospitalario y en UCIN, necesidad de tratamiento farmacológico y su duración.	- Cohortes	AC respecto de UCIN (p<0,001):
2020		- Historia clínica	- <duración de la estancia (13 vs 20 días)
EEUU		- n=105 RN	- <admisión en UCIN (60 vs 100%)
		15 EMPOWER y AC	- <duración estancia en UCIN (3 vs 18 días)
		90 UCIN (control histórico)	- <necesidad de tratamiento farmacológico (37,7 vs 61,5%)
		- Metadona, buprenorfina, heroína	- <duración del tratamiento farmacológico (10 vs 17 días).
		- Escala modificada de Finnegan.	

AC: alojamiento conjunto; CE: cuidado estándar; RN: recién nacido; LA: lactancia artificial; LAbl: lactancia artificial baja en lactosa; LAe: lactancia artificial estándar; LM: lactancia materna; LME: lactancia materna exclusiva; LMx: lactancia mixta; SAN: síndrome de abstinencia neonatal; UCIN: unidad de cuidados intensivos neonatales; n/e: no indica o no especifica.

La lactancia materna fue objeto de cuatro estudios y las dos revisiones, comparada con lactancia artificial (estándar^16^^,^^17^^,^^21^^,^^22^ o baja en lactosa^21^ y mixta^16^^,^^23^). El alojamiento conjunto fue el objeto de seis estudios y una revisión^17^, comparado con cuidado estándar^11^ y UCIN^14^^,^^15^^,^^24^^-^^26^. De ambos tipos de intervenciones se identificó su asociación con los seis resultados indicados en el objetivo de la revisión: duración de la estancia hospitalaria, necesidad y duración de tratamiento farmacológico, severidad de los signos del SAN, admisión en UCIN y reingreso hospitalario tras el alta.

### Lactancia materna

La lactancia materna tuvo un efecto positivo en la *duración del ingreso hospitalario* de los neonatos con SAN. Los alimentados con lactancia materna presentaron ingresos más cortos que los alimentados con leche de fórmula^16^^,^^21^; según Lembeck y col^21^, esta diferencia puede llegar a ser de hasta 7,4 días.

Los neonatos alimentados con leche materna tuvieron menos probabilidades de *necesitar tratamiento farmacológico*^22^, y la *duración del tratamiento* fue inferior que con lactancia artificial^16^^,^^21^ o mixta. En una muestra de 1.738 niños, Favara y col^16^ observaron una reducción en la duración del tratamiento de tres días en neonatos con lactancia materna (exclusiva o mixta) respecto de lactancia artificial.

Las dos revisiones describieron que los bebés que toman leche materna presentaron menor *severidad de los signos* de abstinencia^17^^,^^22^, así como el estudio de McQueen y col^23^, en el que la puntuación en la escala modificada de Finnegan fue 1,6 y 2 puntos inferior en el grupo de lactancia materna que en los de lactancia mixta y lactancia artificial, respectivamente.

La relación entre el tipo de lactancia y la *admisión en UCIN* no fue el objeto de ninguno de los estudios revisados.

Los bebés alimentados con leche materna presentaron una mayor *tasa de reingreso en el hospital* en comparación con el grupo alimentado con leche de fórmula (1,6% vs. 1,3%), sin embargo, esta diferencia no fue estadísticamente significativa^16^.

### Alojamiento conjunto

La *duración de la estancia hospitalaria fue menor en los* neonatos que permanecieron con su madre durante toda la estancia respecto a los ingresados en UCIN^14^^,^^15^^,^^24^^-^^26^, incluso en caso de alojamiento combinado (UCIN y hospitalización con la madre)^24^: Grossman y col observaron 8,5 días de ingreso en el grupo de alojamiento conjunto en planta, significativamente inferior a la de los grupos combinado y UCIN (18 días y 23 días de ingreso). En contraposición con este dato, un estudio canadiense encontró una mayor duración de las estancias hospitalarias en el modelo de alojamiento conjunto respecto del cuidado estándar (17,6 frente a 8,1 días)^11^.

Los bebés mantenidos en alojamiento conjunto tuvieron menos *necesidad de tratamiento farmacológico* para paliar la gravedad de los signos de abstinencia^14^^,^^15^^,^^24^^-^^26^, un 45% de reducción respecto a UCIN según Singh y col^26^.

Varios estudios han descrito una disminución en la *duración del tratamiento farmacológico* cuando se produce alojamiento conjunto en contraste con otros tipos de alojamiento. Estas diferencias oscilan entre 5 y 7 días en la mayoría de los estudios (9,71 vs 15,68 días^14^; 10 vs 17 días^26^; 24 vs 29,5 días^15^).

El estudio de Abrahams y col^11^ no encontró diferencias significativas en la *severidad de los signos de SAN* en neonatos en alojamiento conjunto frente a los que estaban en otro tipo de alojamiento (el 27,3% y el 26,1% presentaron signos graves, respectivamente).

Singh y col^26^ compararon los resultados de *admisión en UCIN* antes de incorporar el modelo de alojamiento conjunto (cuando el 100% de los neonatos con SAN eran ingresados en UCIN por protocolo) con los resultados tras su incorporación (ingreso en UCIN en caso de SAN severo: dos puntuaciones en la escala de Finnegan ≥8 o una >12); el porcentaje de ingreso se redujo al 60%. Un estudio comparó los resultados de *admisión en UCIN* de algunos centros que practicaban el alojamiento conjunto con otros en los que no^11^, encontrando un 15% menos de ingreso en UCIN en los primeros; no se mencionan los criterios empleados para la admisión del neonato en UCIN. La duración de la estancia en UCIN fue entre 2 y 15 días inferior cuando los niños a término habían estado en alojamiento conjunto^11^^,^^14^^,^^26^ (Tabla 2).

El único artículo que estudió la asociación del alojamiento conjunto en la *tasa de reingreso en el hospital*, encontró que 2 de los 60 bebés que habían permanecido en alojamiento conjunto necesitaron ser readmitidos tras el alta para controlar la sintomatología, frente a ninguno de los 50 neonatos ingresados en UCIN (diferencias no significativas)^24^.

## DISCUSIÓN

Los resultados de esta revisión muestran que la lactancia materna y el alojamiento conjunto de la madre con el neonato aportan una serie de beneficios en el abordaje del SAN. El amamantamiento del neonato supone una reducción de la duración de la estancia hospitalaria, de la necesidad de farmacoterapia y de la duración de esta, y de la severidad de los signos del síndrome. Del mismo modo, el alojamiento conjunto conlleva una disminución de la duración de la estancia hospitalaria, de la necesidad de farmacoterapia y de su duración, mostrando también resultados favorables en la disminución del ingreso de los neonatos en UCIN.

Si estas prácticas, de forma aislada, han mostrado un efecto positivo en neonatos con SAN, la combinación de ambas podría suponer incluso un mayor beneficio para esta población.

La tasa de amamantamiento es mayor en los neonatos de las unidades en las que se utilizó el alojamiento conjunto, en comparación con otros sistemas de cuidados^24^. Esto se puede deber a que el alojamiento conjunto favorece la conexión y el apego entre madre e hijo y, por tanto, la práctica de la lactancia materna^15^^,^^24^^,^^26^. Además, este vínculo afectivo resulta imprescindible en las diferentes etapas del desarrollo infantil^27^.

Con respecto a los aspectos menos positivos, algunos estudios muestran que los bebés alimentados con leche materna podrían presentar una mayor probabilidad, aunque no estadísticamente significativa, de ser reingresados en comparación con el grupo alimentado con leche de fórmula^16^, y que el alojamiento conjunto no redujo la severidad de los signos del SAN^11^. Tampoco se puede concluir que el alojamiento conjunto disminuya el reingreso hospitalario^24^, aunque sus autores señalaron que es complejo identificar los motivos de reingreso y que frecuentemente se debe a motivos ajenos al SAN^24^. Un estudio encontró mayor duración del ingreso cuando se realiza alojamiento conjunto, pero los autores no lo relacionaron con el SAN sino con menores recursos socio-económicos de las madres participantes, que necesitaron estar ingresadas más tiempo antes de recibir el alta en condiciones adecuadas para poder cuidar del neonato^11^, algo que su modelo sanitario promueve para apoyarlas tras convertirse en madres y asegurar el bienestar del recién nacido.

Proporcionar cuidados seguros que garanticen el bienestar del recién nacido es responsabilidad de los profesionales de la salud. Los hallazgos de esta revisión muestran que tanto el alojamiento conjunto como la lactancia materna son prácticas beneficiosas para los neonatos con SAN. Sin embargo, estos hallazgos se tienen que contextualizar en cada caso, y esto puede suponer cambios en la manera en la que se atiende en el entorno hospitalario a la madre y al neonato con SAN. Los profesionales suelen mostrar preocupación por dos cuestiones principales: la capacidad de las madres con alguna adicción para cuidar al recién nacido y la necesidad de que el neonato permanezca monitorizado y evaluado frecuentemente^13^^-^^15^. Atender a la primera cuestión supone valorar la capacidad de la madre de cuidar al neonato, y no exclusivamente con pruebas de consumo de sustancias tóxicas, sino que requeriría de pruebas más completas a los progenitores y un trabajo estrecho entre profesionales de la salud y trabajadores sociales. Respecto a la necesidad de que el neonato permanezca monitorizado, se debería resolver promoviendo el alojamiento conjunto en unidades neonatales y UCIN o en otro tipo de unidades en las que se pueda realizar la observación y evaluación del neonato, acompañando de manera continua a las familias.

La morfina y la buprenorfina (los opioides más utilizados en estos estudios) pueden producir depresión respiratoria, además de dependencia y tolerancia^29^, aumentando los riesgos para la salud del neonato. La observación de que la lactancia materna disminuya el número y la duración de los tratamientos farmacológicos es un hallazgo de especial relevancia, ya que gran parte de los fármacos que se utilizan para el tratamiento del SAN no están probados en este grupo poblacional^28^. Sin embargo, proporcionar o no lactancia materna no es algo que deban decidir los profesionales sin contar con la madre, sino que se trata de acompañar a las mujeres valorando la situación e informando adecuadamente para que ellas tomen las decisiones sin prejuicios^30^.

El efecto que el alojamiento conjunto y la lactancia materna tienen en esta reducción de la necesidad de medicación, de los ingresos en las UCIN y en las estancias hospitalarias en general, supone también una reducción de los costes hospitalarios relacionados con el abordaje de este síndrome. Singh y col^26^ han mostrado que el coste diario de la atención de un neonato con SAN se triplica en cuidados intensivos respecto a en una habitación con su familia. Este hecho podría permitir un mejor uso de los recursos, invirtiendo donde pueda ser más necesario para proporcionar una atención de mayor calidad, esto es, en el seguimiento de las familias de manera más adecuada y la monitorización del neonato cerca de su madre.

A pesar de reconocer que la lactancia y el alojamiento conjunto son esenciales para humanizar los cuidados en el nacimiento, no se puede olvidar que no siempre va a ser posible aplicarlos. En el caso de la lactancia materna, puede haber situaciones en las que no se recomiende el amamantamiento, por ejemplo, cuando la madre padece infección por VIH, cuando se encuentra en tratamiento de quimioterapia o radioterapia y cuando la madre es una toxicómana activa^4^. En este último caso habría que tener en cuenta el estado de adicción que presenta la madre para poder aconsejar o no la lactancia materna. Además, tal y como indican varios autores incluidos en esta revisión, en estas situaciones es habitual que los neonatos con SAN vivan en contextos en los que la situación socio-familiar de la madre pueden estar influyendo en aspectos de la salud tanto del neonato como de la propia mujer. Aunque los parámetros incluidos en esta revisión contribuyen a un buen comienzo, centrarse solo en aspectos inmediatos relacionados con la alimentación y el alojamiento conjunto puede ser insuficiente para asegurar el bienestar del niño y de la mujer tras el alta hospitalaria.

Conocer la opinión y deseos de las madres también es esencial a la hora de aconsejar estas medidas; sin embargo, hay que tener en cuenta que sus deseos se pueden ver afectados por numerosos factores. El deseo de amamantar se ve potenciado cuando las mujeres reciben apoyo por parte del personal sanitario, por la percepción favorable de que la leche materna influye en el estado de salud y en el bienestar del neonato, y por la idea de que la LM mejora del vínculo entre ambos^31^. En contraste, el rechazo de la lactancia materna viene motivado por el miedo a que el neonato quede expuesto a sustancias transmitidas a través de la leche, la necesidad de que el neonato tenga que ingresar en UCIN (porque impide permanecer con él el tiempo necesario o la falta de transporte para acudir al hospital tras el alta de la madre), la percepción de la lactancia como una práctica agotadora, el escaso apoyo de los profesionales de la salud o sentimientos de no poder alimentar adecuadamente al neonato por no tener suficiente leche^31^.

El apoyo profesional aparece descrito en la literatura como elemento clave; además, algunos estudios señalan que las necesidades de estas madres para lactar son mayores, al precisar un mayor apoyo en el proceso debido a la inquietud y la hiperfagia que puede presentar un recién nacido con SAN^17^^,^^31^. Por ello, la educación de los padres en el cuidado de los niños con este síndrome es esencial. La mitad de los trabajos revisados^11^^,^^14^^,^^16^^,^^21^^,^^25^^,^^26^ han puesto de manifiesto la relevancia de educar a las familias en aspectos relacionados con el SAN, como los signos y síntomas y las medidas no farmacológicas. Para ello es imprescindible que se impliquen en la práctica del cuidado y participen en las actividades sanitarias junto a los profesionales de la salud^26^. El alojamiento conjunto facilita que esto sea así y, además, el único estudio que midió este aspecto ha mostrado una alta satisfacción en las madres^25^.

Entre las madres participantes de algunos de los estudios revisados^15^^,^^21^^,^^26^ se incluyeron tanto aquellas en tratamiento de deshabituación con metadona o buprenorfina, como aquellas consumidoras de sustancias ilícitas durante el embarazo. Sin embargo, los estudios no muestran análisis diferenciados para ambos tipos de consumo en las variables analizadas en esta revisión. Obtener más información a este respecto podría ayudar a identificar los mejores cuidados para cada situación.

No se puede olvidar que los estudios revisados se centran en el inicio de la vida de los neonatos y no se ha realizado un seguimiento a largo plazo de estos lactantes. Además, los autores no indican si las mujeres estudiadas siguen consumiendo drogas o tratamientos tras el parto. En el caso en que la mujer continúe con lactancia por periodos más largos y siga consumiendo drogas o se presenten recaídas tras su suspensión, se debería trabajar con las madres cómo realizar la suspensión de la lactancia materna. A falta de investigaciones específicas al respecto, cuando ya se ha establecido la lactancia lo recomendable sería aconsejar a las madres retirarla de manera gradual y no repentinamente^30^.

Los resultados de esta revisión son relevantes pero sus hallazgos deben tomarse con cautela al trasladarlos a otros contextos. La principal limitación de esta revisión es que la mayoría de los estudios revisados se realizaron en Norteamérica, por lo que el tipo de droga que prevalece en los estudios analizados son los opioides, mientras que en España y en Europa, el cannabis es la sustancia ilegal más consumida^8^; en futuros estudios, se necesita indagar si existen diferencias en el SAN según el tipo de droga. La localización geográfica también afecta a la atención que se proporciona a los neonatos y, además, las infraestructuras y las prácticas hospitalarias varían de unos estudios a otros, no concretando en la mayoría de los casos a qué se refieren los autores con alojamiento conjunto o siendo el alojamiento conjunto parte de intervenciones más amplias, lo que dificulta la comparación de unas prácticas con otras o indicar cuál de ellas podría tener mejores resultados. Con respecto a los aspectos metodológicos de los estudios incluidos, es importante tener en cuenta que en varios de los estudios revisados se hizo una comparación de los resultados antes de incorporar el alojamiento conjunto con los resultados tras su incorporación^14^^,^^15^^,^^25^^,^^26^ y que no se trata de ensayos clínicos aleatorizados en los que se controlan variables en igualdad de condiciones para ambos grupos. Otra limitación es que la mayoría de los trabajos revisados tienen muestras relativamente pequeñas, si bien esta limitación es relativa porque ha permitido identificar resultados estadísticamente significativos para la mayoría de las variables estudiadas, tal y como se ha podido observar en la presentación de los resultados.

En conclusión, los resultados de esta revisión sugieren que tanto la práctica del alojamiento conjunto como la lactancia materna pueden proporcionar resultados positivos en el abordaje del neonato con SAN. La literatura muestra que estas medidas ayudan a disminuir la medicación administrada para el tratamiento del SAN, el número de días de ingreso hospitalario, la severidad de los signos del síndrome, así como el riesgo de admisión del neonato en UCIN, lo que conllevaría a un mejor uso de los recursos de los centros. Estos recursos se podrían destinar a acompañar al neonato y a su familia educando a los padres e involucrándolos en el cuidado que requiere el neonato, no solo durante la estancia hospitalaria, sino también al alta. Sin embargo, no hay que olvidar que los estudios revisados son escasos y con muestras relativamente pequeñas que se han realizado en contextos diferentes al español, y que existe la necesidad de realizar estudios que aporten este tipo de datos para una mejor aplicabilidad de estas recomendaciones.
